# The safety of laparoscopic D2 distal gastrectomy following neoadjuvant chemotherapy for locally advanced gastric cancer patients: a prospective multicenter trial (CLASS-03a)

**DOI:** 10.1097/JS9.0000000000004531

**Published:** 2025-12-16

**Authors:** Kun Yang, Shen Li, Xuefei Wang, Guoxin Li, Xianli He, Zekuan Xu, Heli Liu, Su Yan, Lu Zang, Gaoping Zhao, Lin Chen, Yong Li, Kuan Wang, Guojun Wang, Weihan Zhang, Yihong Sun, Zhemin Li, Jiang Yu, Tao Wu, Linjun Wang, Jie Ge, Bowen Ho, Bingyu Zuo, Guiqing Jia, Xiaolong Chen, Zhaoqing Tang, Yanfeng Hu, Tao Jin, Deying Kang, Ziyu Li, Jiankun Hu

**Affiliations:** a Department of General Surgery & Laboratory of Gastric Cancer, State Key Laboratory of Biotherapy/Collaborative Innovation Center of Biotherapy and Cancer Center, West China Hospital, Sichuan University, Chengdu, China; b Gastric Cancer Center, West China Hospital, Sichuan University, Chengdu, China; c Department of Gastrointestinal Cancer Center & Key Laboratory of Carcinogenesis and Translational Research (Ministry of Education), Peking University Cancer Hospital, Beijing Institute for Cancer Research, Beijing, China; d Gastric Cancer Center/Department of Gastrointestinal Surgery, Zhongshan Hospital, Fudan University, Shanghai, China; e Department of General Surgery, Nanfang Hospital, Southern Medical University, Guangzhou, China; f Department of General Surgery, Tangdu Hospital, Fourth Military Medical University, Xi’an, China; g Department of General Surgery, The First Affiliated Hospital with Nanjing Medical University, Nanjing, China; h Department of Gastrointestinal Surgery, Xiangya Hospital, Central South University, Changsha, China; i Department of Gastrointestinal Surgery, Affiliated Hospital of Qinghai University, Xining, China; j Department of General Surgery, Ruijin Hospital, Shanghai Jiao Tong University School of Medicine, Shanghai, China; k Department of Gastroenterology, Sichuan Academy of Medical Sciences and Sichuan Provincial People’s Hospital, Chengdu, China; l Department of General Surgery & Institute of General Surgery, Chinese PLA General Hospital First Medical Center, Beijing, China; m Department of General Surgery, Guangdong General Hospital, Guangzhou, China; n Department of Gastrointestinal Surgery, the Cancer Hospital of Harbin Medical University, Harbin, China; o The Department of Gastrointestinal Surgery, The First Affiliated Hospital of Zhengzhou University, Zhengzhou, Henan, China; p Gastric Cancer Center/Department of General Surgery, Zhongshan Hospital, Fudan University, Shanghai, China; q Department of Evidence-based Medicine and Clinical Epidemiology, Sichuan University, Chengdu, China

**Keywords:** clinical trial, gastric cancer, laparoscopic D2 distal gastrectomy, neoadjuvant chemotherapy

## Abstract

**Purpose:**

The objective of this study was to ascertain the safety of laparoscopic distal D2 radical gastrectomy in treating gastric cancer patients after NAC with locally advanced disease (cT3-4a, N0/ +, M0) by evaluating postoperative complications.

**Study Design:**

A prospective, multicenter, single-arm clinical trial.

**Methods:**

This clinical trial was conducted at 14 hospital centers in China. Adults aged 18–75 years with histologically confirmed LAGC (cT3-4a, N0/ +, M0) were enrolled in the study. Participants received three cycles of administration of intravenous oxaliplatin (130 mg/m^2^ on day 1 of each cycle) plus oral capecitabine (1000 mg/m^2^ twice daily on days 1 to 14 of each cycle), repeated every three weeks prior to undergoing laparoscopic distal gastrectomy. The primary endpoint was the postoperative overall morbidity rate. Secondary endpoints included postoperative mortality rate, surgery-related complications, R0 resection rate, and the rate of conversion to laparotomy, response of neoadjuvant chemotherapy (NAC), adverse event rate of NAC, operation time, blood loss, postoperative severe morbidity rate, and postoperative recovery course.

**Results:**

A total of 153 patients who underwent NAC prior to laparoscopic D2 distal gastrectomy were included in the final analysis. The study reported a postoperative overall morbidity rate of 20.9% (95%CI: 15.2%–28.0%), with a postoperative mortality rate of 0%. Pneumonia is the most common complication (9.2%). Ten patients exhibited elevated levels of body fluid amylase without presenting any clinical symptoms or undergoing additional clinical intervention. The R0 resection rate was achieved at 100%. The rate of conversion to laparotomy was 1.3%. 9.2% of patients achieved pathological complete response (pCR) following NAC. The overall incidence of adverse effects after NAC was 20.3% (95%CI: 14.7%–27.3%). The most common grade 3–4 treatment-related adverse events during neoadjuvant treatment were a decrease in platelet count and vomiting, each occurring in 0.7% of patients.

**Conclusion:**

The laparoscopic distal D2 radical gastrectomy demonstrated a favorable safety profile in the treatment of gastric cancer patients with advanced disease (cT3-4a, N0/ +, M0) following NAC.

## Introduction

Gastric cancer exhibits the fourth highest incidence among malignant tumors, with mortality ranking fifth globally^[1]^. In China, the early diagnosis rate of gastric cancer is relatively low, and most patients are diagnosed as locally advanced tumor stage with potentially poor prognosis. The neoadjuvant chemotherapy (NAC) could bring the survival advantage for locally advanced gastric cancer patients (LAGC)^[2]^. The primary goal of NAC is to control the micro-metastasis, and progression of the primary lesion to improve the potential of radical gastrectomy. NAC is recommended for patients with locally advanced stages according to the latest National Comprehensive Cancer Network (NCCN) Gastric Cancer Guidelines^[3]^.

Laparoscopy distal gastrectomy (LDG) can achieve a better postoperative short-term recovery than the traditional open distal gastrectomy (ODG), which can reduce intraoperative blood loss and shorten the postoperative hospital stay^[4]^. Therefore, the Enhanced Recovery After Surgery program of gastric cancer surgery recommends the use of minimally invasive surgery^[5]^. For long-term survival outcomes, there was limited evidence supporting that laparoscopic gastrectomy was comparable to open gastrectomy. Once, due to the lack of high-quality prospective clinical trial results, whether advanced tumors are suitable for laparoscopic surgery is still controversial. Currently, some multi-center prospective randomized controlled trials have been carried out, comparing the safety and long-term survival outcomes between laparoscopic and open gastrectomy in LAGC. CLASS-01 trials reported that for LAGC, laparoscopic D2 distal gastrectomy was safe and feasible^[6]^. Phase III clinical trials for advanced gastric cancer, specifically JLSSG 0901 in Japan and KLASS-02 in Korea^[7,8]^, have been conducted to validate that the extended survival outcomes of laparoscopic surgery are comparable to those of conventional open surgery. Therefore, LDG with D2 lymphadenectomy may be considered as a potential standard treatment option for this patient population^[7,8]^.

This prospective trial follows TITAN Guidelines (2025) for reporting standards and AI transparency^[9]^. The patient’s surgical tolerance and stress response may be inhibited after NAC. Previous studies showed that the presence of edema, effusion, and fibrosis in the perigastric tissue, metastatic lymph nodes, and primary tumor following chemotherapy may pose challenges during tissue dissociation and lymph node dissection in D2 lymphadenectomy for advanced gastric cancers, thereby complicating gastric cancer surgery to some extent^[10]^. One another aspect, the utilization of NAC and laparoscopic surgery has become more prevalent in the management of gastric cancer. Nevertheless, the viability and safety of LDG following NAC remained uncertain. Thus, this trial aimed to confirm the safety of laparoscopy distal D2 radical gastrectomy for the treatment of gastric cancer patients (cT3-4a, N0/ +, M0) after NAC in terms of postoperative complications.

**HIGHLIGHTS**

- This study constitutes the inaugural prospective, multicenter, single-arm trial involving 153 patients across 14 hospitals in China, aimed at evaluating the safety of laparoscopic distal D2 radical gastrectomy for gastric cancer patients following neoadjuvant chemotherapy by assessing postoperative complications.
- Laparoscopic distal D2 radical gastrectomy proved safe for treating advanced gastric cancer (cT3-4a, N0/ +, M0) after NAC.

## Methods

### Study design and participants

This was a prospective, multicenter, open-label, single-arm study, and this trial aimed to evaluate the safety of laparoscopic distal D2 radical gastrectomy for the treatment of locally advanced gastric cancer (cT3-4a, N0/ +, M0) after NAC. This work has been reported in line with Consolidated Standards of Reporting Trials (CONSORT) Guidelines^[11]^.

### Participant eligibility

Between 2018 and 2024, 14 high-volume gastric cancer centers in Mainland China participated in the trial. Consecutive patients who met eligibility criteria were screened at each center.

### Ethical approval and registration:

The study was approved by ethics committees or institutional review boards at participating institutions. All patients provided written informed consent. The registration number is withheld for double-blind review and will be provided upon publication.

### First round assessment

The inclusion criteria of this trial are: (1) aged between 18 and 75 years; (2) primary gastric adenocarcinoma (including pap, tub, muc, sig, and por) confirmed pathologically by endoscopic biopsy; (3) cT3-4a, N0/ +, M0 according to the UICC/AJCC 8th Cancer Staging Manual; (4) without peritoneal metastasis (confirmed by diagnostic laparoscopy and cytology); (5) potentially radical resection (R0) through distal gastrectomy with D2 lymphadenectomy; (6) Eastern Cooperative Oncology Group (ECOG) performance score ≤1; (7) American Society of Anesthesiology (ASA) score ≤3; (8) blood cell count: HB ≥90 g/L, ANC ≥1.5 × 109/L, PLT ≥80 × 109/L; (9) liver and kidney function: BIL <1.5 folds of the upper limit, ALT and AST <2.5 folds of the upper limit, and CREA ≤1 fold of upper limit.

The exclusion criteria are (1) history of upper abdominal surgery (including endoscopic mucosal resection or endoscopic submucosal dissection, but except laparoscopic cholecystectomy); (2) history of acute pancreatitis; (3) enlarged or bulky regional lymph node (maximal diameter >3 cm) by imaging; (4) neoadjuvant therapy prior to eligibility assessment; (5) history of other malignant diseases during the past five years; (6) history of cardiocerebrovascular accident within the past six months; (7) history of continuous systematic administration of corticosteroids within the past month; (8) scheduled simultaneous surgery for other diseases; (9) emergency surgery due to complications of gastric cancer, including bleeding or perforation; (10) outlet obstruction; (11) FEV1 < 50% of predicted value; (12) pregnant or lactating women; (13) severe mental disorders; 14) participation in other clinical studies; 15) refusal to sign the informed consent.

### Second round assessment

After the three cycles of NAC, the second-round assessment would be performed. The tumor response after NAC and the potential of resectability are to be critically evaluated. Seven particular situations were identified: (1) progressive disease (PD) and total gastrectomy required; (2) T4b disease; (3) M1 disease; (4) non-bulky nodes (≥3 cm); (5) intolerance of NAC without complete 3 cycles; (6) completion of 3 cycles but with the presence of severe side effect, ASA score ≥4 and intolerance of surgery; and (7) emergence surgery for bleeding, perforation, or obstruction during NAC.

### Outcomes

The primary outcome was the postoperative overall morbidity rate (during postoperative 30 days). Secondary outcomes included postoperative mortality rate, surgery-related complication, R0 resection rate, the completion rate of laparoscopic surgery, response of NAC, adverse event rate of NAC, operation time, estimated blood loss, postoperative severe morbidity rate, and postoperative recovery course. Tumor regression was evaluated using the tumor regression grading (TRG) system recommended in the NCCN Gastric Cancer Guidelines (2018, version 2, page 22), which was uniformly adopted across all participating centers following consensus meetings, training sessions, and the distribution of standardized pathology manuals and representative reference slides to ensure consistency of interpretation.

### Procedures

Qualified patients after the first round assessment would accept the staging laparoscopy with peritoneal lavage cytology to rule out the P1 or CY1 diseases before the first cycle of NAC. A fixed step-by-step procedure was recommended to prevent missing certain covert spaces^[12]^. Then, patients were administered three cycles of neoadjuvant XELOX therapy. After three cycles of NAC, treatment response was assessed by CT and the second-round assessment was performed. Pathological complete response (pCR) was defined as the absence of viable tumor cells in both the primary gastric lesion and regional lymph nodes (ypT0N0) after completion of NAC. If the tumor response was not progression disease (PD) according to the Response Evaluation Criteria in Solid Tumors (RECIST) criteria version 1.1^[13]^, the surgeries were expected to be standard laparoscopic distal gastrectomy with D2 lymphadenectomy according to the Japanese Gastric Cancer Treatment Guidelines (2014, version 4). Acceptable reconstruction procedures included Billroth-I, Billroth-II with or without Braun, Roux-en-Y, or uncut Roux-en-Y, performed either totally laparoscopically or through a minimal incision. The postoperative complications were recorded and graded according to the Clavien–Dindo classification^[14]^. Postoperative management was standardized across centers, including routine antibiotics, parenteral nutrition, early removal of the nasogastric tube, gradual reintroduction of diet, and drain removal on postoperative day 5 if no complications occurred. Detailed operative procedures and perioperative management are provided in the Supplemental Digital Content Material, available at: http://links.lww.com/JS9/G389

### Adjuvant treatment

Those who receive NAC and LDG-D2 procedure would be suggested to adjuvant chemotherapy, with limitation of regimen, but commonly XELOX regimen was preferred if NAC response was non-PD according to the RECIST criteria version 1.1^[13]^. Pathological tumor regression grade was not mandatory for regimen decision. Adjuvant radiotherapy was acceptable, and better to be performed in a multidisciplinary manner. Palliative chemotherapy would be considered in situations concerning distal metastasis, recurrence, or progression to unresectable disease.

### Quality control

All candidate surgeons in this trial were selected from the members of the CLASS group, who met the following criteria: (1) performed at least 50 LDG-D2 surgeries; (2) performed at least 300 gastrectomies for gastric cancer patients annually in each institute; (3) performed both open and laparoscopic D2 gastrectomy; (4) verified by the CLASS-03a academic committee through peer-review of unedited videos of their both open and laparoscopic D2 gastrectomies.

Among the operated patients, five operative photos were mandatory to be collected and stored in the PI center. The five photos were used for verification on the standard D2 lymphadenectomy, including (1) inferior pylorus area; (2) left gastroepiploic vessel stump site; (3) right-side area superior to the pancreas; (4) left-side area superior to the pancreas; (5) right-side cardia and residual lesser curvature. In addition, the pictures of incision and specimens were required to be collected.

### Sample size

The sample size calculation was based on published literature and preliminary data, which reported a postoperative complication rate of approximately 23% for distal gastrectomy following NAC. A non-inferiority margin of 35% was prespecified, as rates above this threshold would not be clinically acceptable. Using a one-sided α of 0.05 and β of 0.20 (power = 80%), the required sample size was estimated to be 95 patients.

To account for attrition, a dropout rate of 42.5% was anticipated, including 37.5% of patients unable to complete three cycles of NAC and 5% who might decline surgery or progress to unresectable disease. Accordingly, the target enrollment was set at 166 patients, of whom 153 were ultimately included in the final analysis.

### Statistical analysis

Descriptive statistics were conducted on baseline, clinicopathological characteristics, and adverse effects after NAC, and postoperative complications. Continuous variables with a normal distribution were reported as mean (standard deviation), while non-normal variables were presented as median (minimum to maximum range). Categorical variables were expressed as numbers (percentages). The proportion of patients exhibiting various responses was calculated using the Clopper–Pearson method, with a 95% confidence interval (CI). The R software version 4.0.3 (R Foundation for Statistical Computing, Vienna, Austria) was used for all analyses.

Data completeness was prospectively monitored across all participating centers. Key study endpoints, including postoperative morbidity, mortality, and R0 resection rate, were available for all patients; therefore, no imputation was required for the primary outcome analysis. For secondary variables with occasional missing entries (e.g., laboratory tests or perioperative parameters), analyses were performed on a complete-case basis. Given the minimal proportion of missing data, multiple imputation or sensitivity analyses were not performed.

## Results

### Characteristics of patients

**Figure 1.**
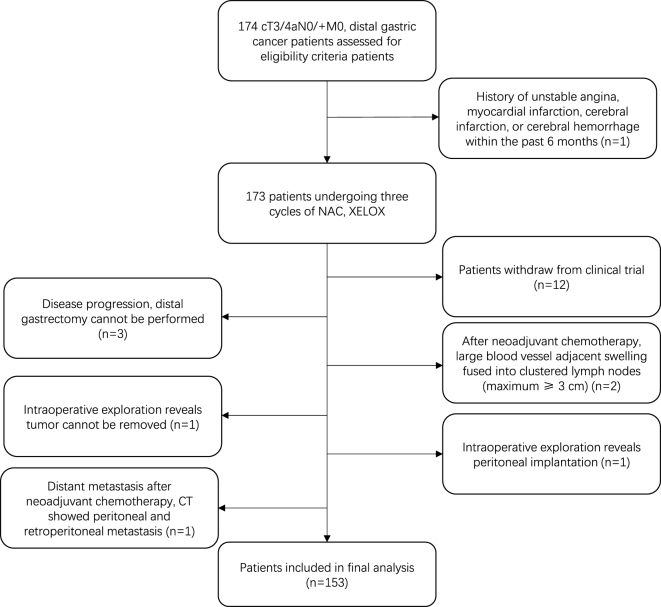
The flow diagram of the study process.

Between September 2018 and January 2024, 153 patients from 14 institutions were enrolled in this study. The flow diagram of the study process is shown in Figure 1. Male patients accounted for 79.1% (121/153) of the included patients. The median age and body mass index of patients were 59.00 [52.00, 67.00] years, and 22.72 [21.48, 25.39] kg/m^2^, respectively. All patients had a score of < 2 in the ECOG performance status classification. The distribution of cases based on clinical staging [American Joint Committee on Cancer (AJCC) 8th Edition) is as follows: cT3N0M0 11 (7.2%), cT3N + M0 58 (37.9%), cT4aN0M0 9 (5.9%), and cT4aN + M0 75 (49.0%). The majority (85.0%) of cases of gastric cancer are found in the lower third of the stomach. The detailed information on the included patients is shown in Table 1.

**Table 1:** The basic characteristics of gastric cancer patients.

	Level	Overall
Number		153
Sex (%)	Male	121 (79.1)
	Female	32 (20.9)
Age (median [IQR])		59.00 [52.00, 67.00]
BMI (median [IQR])		22.72 [21.48, 25.39]
Tumor size (median [IQR])		4.00 [3.00, 5.00]
History of surgery (%)	No	128 (83.7)
	Yes	25 (16.3)
Previous comorbidities (%)	No	85 (55.6)
	Yes	68 (44.4)
ECOG (%)	0	95 (62.1)
	1	58 (37.9)
ASA (%)	I	49 (32.0)
	II	101 (66.0)
	III	3 (2.0)
Tumor location (%)	L	130 (85.0)
	LM	8 (5.2)
	M	15 (9.8)
cTNM (AJCC 8th)	cT3N0M0	11 (7.2)
	cT3N + M0	58 (37.9)
	cT4aN0M0	9 (5.9)
	cT4aN + M0	75 (49.0)
Response evaluation by CT after NAC (%)	CR	7 (4.6)
	Non-CR	146 (95.4)
	PD	1 (0.7)
	Non-PD	152 (99.3)

AJCC, The American Joint Committee on Cancer; ASA, American society of Anesthesiologists; BMI, body mass index; ECOG, Eastern Cooperative Oncology Group; IQR, interquartile range; L, lower one-third; M, middle one-third; NAC, neoadjuvant chemotherapy.

### NAC findings

All patients accepted NAC administered by three cycles of XELOX NAC. 31 (20.3%) individuals had adverse reactions, 29 patients had grade 1 and 2 adverse reactions, and the remaining 2 patients had grade 3–4 adverse reactions. The most frequent AEs among patients are anemia (3.9%), limb numbness (3.9%), platelet count decrease (3.3%), white blood cell count decrease (2.0%), and vomiting (2.0%). Other AEs included hepatic dysfunction (1.3%), diarrhea (0.7%), palpitation (0.7%), hypoalbuminemia (0.7%), and phlebitis (0.7%). The therapeutic response rates for complete CR and non-CR following NAC were 4.6% and 95.4%, respectively. Similarly, the rates for PD and non-PD were 0.7% and 99.3%, respectively. The results of adverse effects were shown in Table 2.

**Table 2:** The adverse effects of NAC.

	Level	Overall	Grade 3–4
*n*		31 (20.3)	
AEs (%)	Nausea	2 (1.3)	0
	Vomiting	3 (2.0)	1 (0.7)
	Diarrhea	1 (0.7)	0
	Hepatic dysfunction	2 (1.3)	0
	Anemia	6 (3.9)	0
	Hypoalbuminemia	1 (0.7)	0
	Palpitation	1 (0.7)	0
	Phlebitis	1 (0.7)	0
	Limb numbness	6 (3.9)	0
	White blood cell count decrease	3 (2.0)	0
	Platelet count decrease	5 (3.3)	1 (0.7)

### Operative outcomes and pathological results

The operative outcomes are summarized in Table 3. All patients underwent distal radical gastrectomy with D2 lymphadenectomy. Of the 153 patients, 151 (98.7%) underwent LDG, while two patients required conversion to open gastrectomy. Reconstruction methods included B-I, B-II, and B-II with Braun, Roux-en-Y, and Uncut Roux-en-Y in proportions of 2.0%, 51.0%, 37.9%, 8.5%, and 0.7%, respectively. The median number of lymph nodes dissected was 22 [15.00–31.00]. The rate of pCR was 9.2%, and the distribution of grade 1, grade 2, and grade 3 after neoadjuvant chemotherapy was 39 (25.5%), 80 (56.8%), and 13 (8.5%), respectively.

**Table 3:** The information of surgery and pathology for gastric cancer patients.

	Level	Overall
Number		153
Operation time (mean (SD))		267.50 (70.04)
Laparoscopic (%)	Laparoscopic	151 (98.7)
	Laparoscopic conversion to open	2 (1.3)
Ascites (%)	No	137 (89.5)
	Yes	16 (10.5)
Degree of fibrosis^a^ (%)	Grade 0	11 (7.2)
	Grade 1	64 (41.8)
	Grade 2	72 (47.1)
	Grade 3	6 (3.9)
Degree of edema^b^ (%)	Grade 0	28 (18.3)
	Grade 1	83 (54.2)
	Grade 2	40 (26.1)
	Grade 3	2 (1.3)
Degree of effusion^c^ (%)	Grade 0	34 (22.2)
	Grade 1	81 (52.9)
	Grade 2	36 (23.5)
	Grade 3	2 (1.3)
Gastrectomy (%)	DG	153 (10.0)
Lymph node dissection (%)	D2	133 (86.9)
	D2 +	20 (13.1)
Reconstruction (%)	B-I	3 (2.0)
	B-II	78 (51.0)
	B-II + Braun	58 (37.9)
	Roux-en-Y	13 (8.5)
	Uncut Roux-en-Y	1 (0.7)
Radical surgery (%)	R0	153 (100.0)
Borrman (%)	III	90 (58.8)
	II	53 (34.6)
	IV	7 (4.6)
	I	3 (2.0)
Grade (%)	G1	3 (2.0)
	G2	32 (20.9)
	G3	89 (58.2)
	G4	1 (0.7)
	Gx	28 (18.3)
ypT (%)	T0	14 (9.2)
	T1a	5 (3.3)
	T1b	16 (10.5)
	T2	38 (24.8)
	T3	46 (30.1)
	T4a	32 (20.9)
	T4b	2 (1.3)
ypN (%)	N0	92 (60.1)
	N1	32 (20.9)
	N2	11 (7.2)
	N3a	13 (8.5)
	N3b	5 (3.3)
Number of lymph nodes dissection (median [IQR])		22.00 [15.00, 31.00]
Blood loss (median [IQR])		50.00 [40.00, 100.00]
Time to first flatus (median [IQR])		3.00 [2.00, 4.00]
Tumor nodules (%)	1	1 (0.7)
	2	1 (0.7)
	0	151 (98.7)
M (%)	0	153 (100.0)
Vascular invasion (%)	Positive	27 (17.6)
	Negative	126 (82.4)
Nerve invasion (%)	Positive	35 (23.2)
	Negative	116 (76.8)
Pancreatic membranes invasion (%)	Negative	134 (87.6)
	NA	19 (12.4)
Transverse colon membranes invasion (%)	Positive	13 (8.5)
	Negative	121 (79.1)
	NA	19 (12.4)
Lymphatic vessel invasion (%)	Positive	22 (14.4)
	Negative	94 (61.4)
	NA	37 (24.2)
Tumor regression score (%)	Grade 0	14 (9.2)
	Grade 1	39 (25.5)
	Grade 2	87 (56.8)
	Grade 3	13 (8.5)

IQR, interquartile range; NA, not available; SD, standard deviation.

^a^ Four categories of fibrosis: grade 0, no fibrosis; grade 1, slight fibrosis with threadiness fibrous bands, clear dissecting space could be found between the fibrous tissues and adventitia/normal tissues; grade 2, moderate fibrosis with flaky fibrous tissues, the difficulty of tissue and lymph nodes dissection increased although dissecting space could be found between the fibrous tissues and adventitia/normal tissues; grade 3, severe fibrosis with hard and flaky fibrous membrane, the difficulty of tissue and lymph nodes dissection increased extremely and the fibrous tissues merges with adventitia/normal tissues without dissecting space^[15]^.

^b^ Four categories of edema: grade 0, no obvious tissue edema; grade 1, slight tissue edema and swelling, no obvious effusion when dissecting the capsule of connective tissues; grade 2, moderate tissue edema and swelling, a few effusion when dissecting the capsule of connective tissues; grade 3, severe tissue edema and swelling with high tension on the capsule of connective tissues, tension blister could be observed in some patients, continuous effusion when dissecting the capsule of connective tissues^[15]^.

^c^ Four categories of intraoperative effusion: grade 0, no obvious effusion; grade 1, slight effusion and a few intraperitoneal exudations; grade 2, moderate effusion and continuous intraperitoneal exudation necessitating interrupted suction; grade 3, severe effusion and continuous intraperitoneal exudation necessitating constant suction^[15]^.

### Postoperative morbidity and mortality

**Figure 2.**
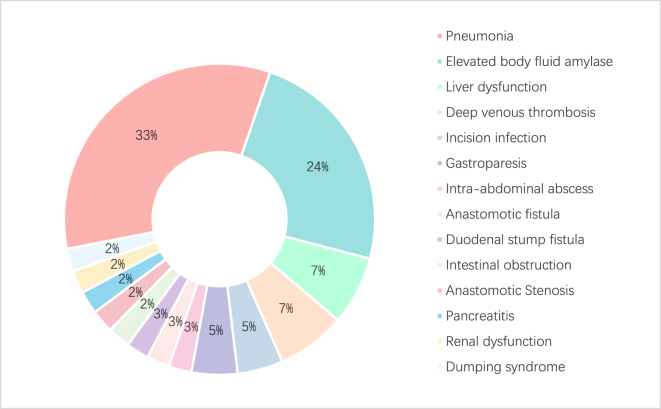
Distribution of surgery-related complications

The research findings indicated a postoperative overall morbidity rate of 20.9% (95%CI: 15.2%–28.0%), with a postoperative mortality rate of 0%. The rate of surgery-related complications was reported at 4.6% (95%CI: 2.2%–9.2%). Complications were assessed and graded according to the Clavien–Dindo classification within 30 days postoperatively. The majority of events were Clavien–Dindo grade I–II and resolved with conservative management. Pneumonia, the most common complication (9.2%), typically occurred within the first postoperative week and was managed with antibiotics and respiratory physiotherapy. Gastrointestinal complications such as anastomotic fistula, duodenal stump fistula, and intestinal obstruction were rare (≤1.0% each) and were successfully treated with drainage, nutritional support, or decompression without the need for reoperation. Elevated drain amylase levels (6.5%) were biochemical findings without clinical sequelae. Re-hospitalization occurred in 10 patients (6.5%) within 30 days, primarily due to incision infection and deep vein thrombosis. All readmitted patients recovered after appropriate medical management, and no patient required intensive care or reoperation. Postoperative complications are presented in Table 4, and the distribution of surgery-related complications was shown in Figure 2.

**Table 4:** The results of postoperative complications.

	Level	Overall
*n*		153
Overall morbidity rate (30 days)	No	121 (79.1)
	Yes	32 (20.9)
Surgery-related postoperative complications (30 days)	No	146 (95.4)
	Yes	7 (4.6)
Incision infection	Yes	2 (1.3)
Intra-abdominal abscess	Yes	1 (0.7)
Anastomotic fistula	Yes	1 (0.7)
Duodenal stump fistula	Yes	1 (0.7)
Intestinal obstruction	Yes	1 (0.7)
Anastomotic stenosis	Yes	1 (0.7)
Gastroparesis	Yes	2 (1.3)
Pancreatitis	Yes	1 (0.7)
Pneumonia	Yes	14 (9.2)
Renal dysfunction	Yes	1 (0.7)
Liver dysfunction	Yes	3 (2.0)
Deep venous thrombosis	Yes	3 (2.0)
Dumping syndrome	Yes	1 (0.7)
Elevated body fluid amylase	Yes	10 (6.5)
Reoperation (%)	No	153 (100.0)
Intensive care unit (%)	No	153 (100.0)
Re-hospital (%)	No	143 (93.5)
	Yes	10 (6.5)

## Discussion

This trial provided evidence of the safety of LDG for LAGC following NAC. It represented the first prospective, multicenter, single-arm trial involving 153 patients across 14 hospitals in China that assessed the safety of laparoscopic distal D2 radical gastrectomy in treating gastric cancer patients after NAC by evaluating postoperative complications. Unlike CLASS-01 and KLASS-02, which enrolled treatment-naïve patients, our trial specifically evaluated the safety of laparoscopic D2 distal gastrectomy after NAC. This represents a clinically distinct population, as NAC introduces surgical challenges such as fibrosis and tissue edema. Our results demonstrate that despite these challenges, morbidity remained low and R0 resection rates were 100%. Thus, our study complements prior RCTs by extending the evidence base to the post-NAC setting, which is increasingly relevant in clinical practice.

There is an emerging trend in the use of NAC in patients with locally advanced gastric adenocarcinoma, with the potential to reduce tumor staging, improve the effectiveness of surgical procedures, suppress tumor cell micro-metastasis, decrease the risk of tumor metastasis, and enhance survival rates. According to the guidelines established by the NCCN^[3]^ for the management of gastric cancer, NAC (evidentiary Category 1) is preferred as the optimal therapeutic approach for LAGC. The RESOLVE trial^[15]^, which assessed the effectiveness and safety of perioperative and postoperative S-1 and oxaliplatin (SOX) in comparison with postoperative capecitabine and oxaliplatin (CapOx) in patients with locally advanced gastric cancer undergoing D2 gastrectomy, revealed that neutropenia was the most common grade 3–4 adverse event. Specifically, 32 (12%) of 258 patients in the adjuvant-CapOx group, 21 (8%) of 249 patients in the adjuvant-SOX group, and 30 (10%) of 310 patients in the perioperative-SOX group experienced neutropenia. The phase 3 RESONANCE trial conducted an investigation into the effectiveness and safety of the S-1 plus oxaliplatin (SOX) regimen as a perioperative chemotherapy treatment for gastric cancer. The predominant adverse events observed were thrombocytopenia and fatigue, with neutropenia being the most commonly reported severe adverse event^[16]^. The findings of our study indicated that 31 individuals (20.3%) experienced adverse reactions, with 2 patients exhibiting grade 3–4 adverse events when accepting NAC, and all patients completed the surgery within the prescribed time after receiving NAC, with a 9.2% rate of pCR that showed the XELOX as a neoadjuvant regimen was feasible.

Additionally, laparoscopic surgery demonstrates potential as a therapeutic option for locally advanced gastric cancer by effectively mitigating surgical trauma and facilitating postoperative recovery, bolstered by increasing number of studies confirming its safety and effectiveness. Nevertheless, there remains a lack of high-quality evidence regarding the combined use of neoadjuvant chemotherapy and laparoscopic distal gastrectomy for this condition. Conducting a trial to investigate this combination would present a challenging endeavor that could potentially yield new insights and modifications to the current treatment paradigm for LAGC.

There is a potential for a higher incidence of surgical complications following neoadjuvant therapy because NAC has the potential to exacerbate tissue fibrosis in the vicinity of tumors and metastatic lymph nodes, leading to indistinct anatomical demarcations. Furthermore, NAC-induced tissue edema may complicate the recognition of anatomical landmarks by causing swelling. The effusion of tissue fluid following NAC administration can obscure the surgical field, while the heightened vulnerability of healthy tissues may contribute to increased bleeding tendencies, prolonged surgical durations, and difficulties in accurately delineating tumor margins during surgery^[17]^. Furthermore, the adverse effects of chemotherapy result in a decrease in surgical tolerance. The above factors augment the complexity of surgical procedures and heighten the likelihood of postoperative complications. However, the efficacy of these findings may be mitigated by the inherent benefits of laparoscopic surgery, including improved exposure and visual magnification, facilitating precise manipulation of organs, blood vessels, and nerves during LDG. Furthermore, the utilization of sophisticated tools such as a Harmonic scalpel and Ligasure during LDG may contribute to minimizing the harm inflicted on healthy tissues during surgery, thereby decreasing the occurrence of adverse events^[18]^. One retrospective study including 153 patients accepted NAC conducted at our center examined the incidence of postoperative complications in two groups (LAG: 18.2% vs. OG: 30.3%, p = 0.120), finding no statistically significant difference between them. Another trial including 96 LAGC patients in China^[19]^ concluded that LADG may provide advantages in terms of improved postoperative safety and tolerance to adjuvant chemotherapy when compared to ODG in patients with LAGC who have undergone NAC (20% vs 46%; P = .007). A randomized trial was conducted in thirteen European hospitals involving 96 patients who underwent NAC^[20]^. The study found no statistically significant disparity in postoperative complications between patients who underwent open total gastrectomy (OTG) and those who underwent minimally invasive total gastrectomy (MITG), with complication rates of 42.9% and 34.0%, respectively (*P* = 0.408). Regarding postoperative complications, our research revealed an overall postoperative morbidity rate of 20.9% (95%CI: 15.2%–28.0%), with no instances of postoperative mortality which was consistent with previous studies. Though the dissection of lymph nodes in the study (22.00 [15.00, 31.00]) was relatively small, which exceeds the AJCC recommendation of a minimum of 16 nodes for accurate staging. This inadequacy may be attributed to the reduction in size of lymph nodes due to neoadjuvant chemotherapy, rendering them difficult to detect during surgery. Although the node yield was somewhat lower than that reported in some trials involving treatment-naïve patients (e.g., CLASS-01, KLASS-02), this likely reflects chemotherapy-related histological changes rather than inadequate dissection. Importantly, the uniformly high R0 resection rate (100%) supports the oncologic adequacy of surgery in this cohort. Nevertheless, the impact of lymph node yield on long-term survival and staging precision in the post-NAC setting warrants further investigation, and these outcomes will be addressed in future follow-up analyses. In general, in the management of patients with LAGC, laparoscopic gastrectomy after NAC could be considered a favorable safety profile and feasibility across multiple centers.

This study is subject to various constraints. First, the single-arm, open-label design without a randomized control group limits causal inference and precludes direct comparisons with open surgery or alternative regimens. Second, surgeons were involved in both patient selection and outcome assessment, which may introduce observer bias, although this was mitigated by standardized eligibility criteria and the use of objective scoring systems such as the Clavien–Dindo classification, CTCAE, and predefined TRG criteria. Third, although this was a multicenter trial involving 14 institutions, some centers contributed relatively few patients, which restricted the ability to perform reliable inter-site comparisons. Fourth, while the overall morbidity rate was reported with a 95% CI, the relatively small number of events limited the feasibility of robust multivariable or predictive analyses. Fifth, all patients were treated in China, which may restrict the generalizability of the findings to other populations and healthcare systems. Sixth, only the XELOX regimen was employed as neoadjuvant chemotherapy, which may not reflect practices in regions where alternative regimens such as SOX or FLOT are standard; further evaluation of different NAC strategies, including immunotherapy-based approaches, is warranted. Furthermore, survival outcomes such as OS and DFS were not yet mature at the time of analysis, and longer follow-up is required to establish the long-term oncologic efficacy of laparoscopic distal gastrectomy after NAC. Finally, this study was designed as a prospective, multicenter, single-arm trial primarily to evaluate the safety and feasibility of laparoscopic D2 distal gastrectomy following neoadjuvant chemotherapy in patients with locally advanced gastric cancer. While this pragmatic design enabled us to generate real-world safety data under standardized surgical protocols, the lack of a control group and randomization inevitably limits the ability to establish causal inferences or to directly compare laparoscopic with open approaches. Therefore, the findings should be interpreted with caution and regarded as descriptive evidence of safety rather than conclusive proof of superiority. To overcome this limitation, future randomized controlled trials, including our planned CLASS-03b study, are warranted to provide higher-level evidence regarding both perioperative and long-term oncologic outcomes.

## Clinical implication

In the context of clinical decision-making, the present findings warrant further consideration. The overall morbidity rate of 20.9% and the pneumonia rate of 9.2% were comparable to those reported in previous multicenter trials of laparoscopic gastrectomy and remain below the thresholds typically regarded as acceptable for major abdominal surgery, thereby supporting the perioperative safety of LDG even following NAC. The observed pCR rate of 9.2% with the XELOX regimen is consistent with previously reported data, reinforcing its feasibility and compatibility with subsequent minimally invasive surgery. Moreover, the incidence of chemotherapy-related adverse events (20.3%) was manageable, with very few grade 3–4 toxicities, indicating that NAC can be safely integrated into perioperative treatment pathways without substantially compromising surgical outcomes. Taken together, these results provide clinically relevant evidence that LDG after NAC is feasible and oncologically sound, although long-term survival data remain necessary before firm recommendations can be made.

## Conclusion

The laparoscopic distal D2 radical gastrectomy was safe and effective for treating advanced gastric cancer patients who received neoadjuvant chemotherapy.

## Data Availability

The datasets generated and/or analyzed during the current study are not publicly available due to patient privacy and institutional policy restrictions, but de-identified data may be made available from the corresponding author on reasonable request and with approval from the institutional review board.

## Research registration unique identifying number (UIN)

This was a prospective, multicenter, open-label, single-arm study (ClinicalTrials.gov Identifiers: NCT03468712).
